# San Antonio Statement on Brominated and Chlorinated Flame Retardants

**DOI:** 10.1289/ehp.1003089

**Published:** 2010-10-28

**Authors:** Joseph DiGangi, Arlene Blum, Åke Bergman, Cynthia A. de Wit, Donald Lucas, David Mortimer, Arnold Schecter, Martin Scheringer, Susan D. Shaw, Thomas F. Webster

**Affiliations:** 1 International POPs Elimination Network, Berkeley, California, USA; 2 Department of Chemistry, University of California, Berkeley, California, USA; 3 Green Science Policy Institute, Berkeley, California, USA; 4 Department of Materials and Environmental Chemistry, and; 5 Department of Applied Environmental Science, Stockholm University, Stockholm, Sweden; 6 Lawrence Berkeley National Laboratory, Berkeley, California, USA; 7 Food Standards Agency, London, United Kingdom; 8 University of Texas School of Public Health, Dallas, Texas, USA; 9 Institute for Chemical and Bioengineering, ETH Zürich, Zürich, Switzerland; 10 Marine Environmental Research Institute, Center for Marine Studies, Blue Hill, Maine, USA; 11 Department of Environmental Health, Boston University School of Public Health, Boston, Massachusetts, USA

We, scientists from a variety of disciplines, declare the following:

1. Parties to the Stockholm Convention have taken action on three brominated flame retardants that have been listed in the treaty for global elimination. These substances include components of commercial penta-bromodiphenyl ether and commercial octa-bromodiphenyl ether, along with hexabromobiphenyl. Another brominated flame retardant, hexabromocyclododecane, is under evaluation.
2. Many commonly used brominated and chlorinated flame retardants  can undergo long-range environmental transport.
3. Many brominated and chlorinated flame retardants appear to be persistent and bioaccumulative, resulting in food chain contamination, including human milk.
4. Many brominated and chlorinated flame retardants lack adequate toxicity information, but the available data raises concerns.
5. Many different types of brominated and chlorinated flame retardants have been incorporated into products even though comprehensive toxicological information is lacking.
6. Brominated and chlorinated flame retardants present in a variety of products are released to the indoor and outdoor environments.
7. Near-end-of-life and end-of-life electrical and electronic products are a growing concern as a result of dumping in developing countries, which results in the illegal transboundary movement of their hazardous constituents. These include brominated and chlorinated flame retardants.
8. There is a lack of capacity to handle electronic waste in an environ-mentally sound manner in almost all developing countries and countries with economies in transition, leading to the release of hazardous substances that cause harm to human health and the environment. These substances include brominated and chlorinated flame retardants.
9. Brominated and chlorinated flame retardants can increase fire toxicity, but their overall benefit in improving fire safety has not been proven.
10. When brominated and chlorinated flame retardants burn, highly toxic dioxins and furans are formed.
  Therefore, these data support the following:
11. Brominated and chlorinated flame retardants as classes of  substances are a concern for persistence, bioaccumulation, long-range transport, and toxicity.
12. There is a need to improve the availability of and access to information on brominated and chlorinated flame retardants and other chemicals in products in the supply chain and throughout each product’s life cycle.
13. Consumers can play a role in the adoption of alternatives to harmful flame retardants if they are made aware of the presence of the substances, for example, through product labeling.
14. The process of identifying alternatives to flame retardants should include not only alternative chemicals but also innovative changes in the design of products, industrial processes, and other practices that do not require the use of any flame retardant.
15. Efforts should be made to ensure that current and alternative chemical flame retardants do not have hazardous properties, such as mutagenicity and carcinogenicity, or adverse effects on the reproductive, developmental, endocrine, immune, or nervous systems.
16. When seeking exemptions for certain applications of flame retardants, the party requesting the exemption should supply some information indicating why the exemption is technically or scien-tifically necessary and why potential alternatives are not technically or scientifically viable; a description of potential alternative processes, products, materials, or systems that eliminate the need for the chemical; and a list of sources researched.
17. Wastes containing flame retardants with persistent organic pollutant (POP) characteristics, including products and articles, should be disposed of in such a way that the POP content is destroyed or irreversibly transformed so that they do not exhibit the charac-teristics of POPs.
18. Flame retardants with POP characteristics should not be permitted to be subjected to disposal operations that may lead to recovery, recycling, reclamation, direct reuse, or alternative uses of the substances.
19. Wastes containing flame retardants with POP properties should not be transported across international boundaries unless it is for disposal in such a way that the POP content is destroyed or  irreversibly transformed.
20. It is important to consider product stewardship and extended  producer responsibility aspects in the life-cycle management of products containing flame retardants with POP properties, including electronic and electrical products.

## Signatories

**San Antonio Statement on Brominated and Chlorinated Flame Retardants**

Signatories as of publication date. Institutions are for identification purposes only.

**Sam Adu-Kumi**, M.S., Deputy Director, Environmental Protection Agency, Accra, Ghana

**Björn Albinson**, Fire Protection Engineer (retired), Karlstad, Sweden

**Henrik Alm**, M.S., Doctoral Student, Pharmaceutical Biosciences, Division of Toxicology, Uppsala University, Uppsala, Sweden

**Misha Askren**, M.D., F.A.A.F.P., Physician, Southern California Permanente Medical Group, Pasadena, CA, USA

**Ralph Baker**, M.S., Ph.D., Chief Scientist, TerraTherm Inc., Fitchburg, MA, USA

**John Balmes**, M.D., Professor of Medicine, University of California, San Francisco, San Francisco, CA, USA, and Professor of Environmental Health Sciences, University of California, Berkeley, Berkeley, CA, USA

**Scott Bartell**, Ph.D., Assistant Professor, University of California, Irvine, Irvine, CA, USA

**Georg Becher**, Ph.D., Department Director and Professor, Analytical Chemistry, Norwegian Institute of Public Health, Oslo, Norway

**David C. Bellinger**, Ph.D., Professor, Harvard Medical School and Harvard School of Public Health, Boston, MA, USA

**Stephen Bent**, M.D., Associate Professor of Medicine, Psychiatry, Epidemiology & Biostatistics, University of California, San Francisco, San Francisco, CA, USA

**Åke Bergman**, Ph.D., Professor, Environmental Chemistry, Stockholm University, Stockholm, Sweden, and Board Member, International Panel on Chemical Pollution, Zürich, Switzerland

**Anders Bignert**, Ph.D., Professor, Contaminant Research, Swedish Museum of Natural History, Stockholm, Sweden

**Justina Björklund**, M.S., Graduate Student, Applied Environmental Science, Stockholm University, Stockholm, Sweden

**Arlene Blum**, Ph.D., Visiting Scholar, Chemistry, University of California, Berkeley, Berkeley, CA, USA

**Christian Bogdal**, Ph.D., Researcher, Swiss Federal Institute of Technology, Zürich, Switzerland

**Phil Brown**, Ph.D., Professor, Sociology and Environmental Studies, Brown University, Providence, RI, USA

**David Camann**, M.S., Staff Scientist, Southwest Research Institute, San Antonio, TX, USA

**Carmela Centeno**, M.S., Ph.D., Industrial Development Officer, United Nations Industrial Development Organization, Vienna, Austria

**Ibrahim Chahoud**, Ph.D., Professor of Reproductive Toxicology, Institute of Clinical Pharmacology and Toxicology, Charité-Universitätsmedizin Berlin, Berlin, Germany

**Eliza Chin**, M.D., M.P.H., President, American Medical Women’s Association, Philadelphia, PA, USA

**Brock Chittim**, M.S., General Manager, Wellington Laboratories, Guelph, Ontario, Canada

**Carsten Christophersen**, Ph.D., Associate Professor, Chemistry, University of Copenhagen, Copenhagen, Denmark

**Bradley Clarke**, Ph.D., Research Fellow, Imperial College, London, United Kingdom

**Theo Colborn**, Ph.D., Professor Emeritus, University of Florida, Gainesville, Florida, USA

**Kathleen Collins**, Ph.D., Professor, Molecular and Cell Biology, University of California, Berkeley, Berkeley, CA, USA

**Terrence Collins**, Ph.D., Teresa Heinz Professor of Green Chemistry and Director of the Institute for Green Science, Carnegie Mellon University, Pittsburgh, PA, USA

**Adrian Covaci**, Ph.D., Professor, University of Antwerp, Antwerp, Belgium

**Craig Criddle**, Ph.D., Professor, Civil and Environmental Engineering, Stanford University, Palo Alto, CA, USA

**Margarita Curras-Collazo**, Ph.D., Associate Professor, Cell Biology and Neuroscience, University of California, Riverside, Riverside, CA, USA

**Kyle D’Silva**, Ph.D., Product Manager, Thermo Fisher Scientific, Dreieich, Germany

**Devra Davis**, M.A., Ph.D., M.P.H, Visiting Professor, Georgetown University, Washington, DC, USA, and Founder, Environmental Health Trust, Teton Village, WY, USA

**Joao De Assuncao**, M.S., Ph.D., Professor and Department Head, Environmental Health, University of Sao Paulo School of Public Health, Sao Paulo, Brazil

**Cynthia A. de Wit**, Ph.D., Professor, Applied Environmental Science, Stockholm University, Stockholm, Sweden

**Mike Denison**, Ph.D., Professor of Environmental Toxicology, University of California, Davis, Davis, CA, USA

**Miriam Diamond**, Ph.D., Professor, Geography, University of Toronto, Toronto, Ontario, Canada

**Joseph DiGangi**, Ph.D., Senior Scientist and Technical Advisor, International POPs Elimination Network, Berkeley, CA, USA

**Alin Dirtu**, Ph.D., Researcher, University of Antwerp, Antwerp, Belgium

**Michelle Douskey**, Ph.D., Lecturer, Chemistry, University of California, Berkeley, Berkley, CA, USA

**Anne Ehrlich**, Ph.D., Senior Research Scientist, Biology, Stanford University, Palo Alto, CA, USA

**David Epel**, Ph.D., Jane & Marshall Steel Jr. Professor Emeritus in Marine Sciences, Cell and Developmental Biology, Stanford University, Palo Alto, CA, USA

**Brenda Eskenazi**, M.A., Ph.D., Jennifer and Brian Maxwell Professor of Maternal Health and Epidemiology, University of California, Berkeley, Berkeley, CA, USA

**Tim Evans**, Ph.D., Cancer Registration Information Manager, West Midlands Cancer Intelligence Unit, Birmingham, United Kingdom

**Peter Fantke**, Ph.D., Research Associate, Institute of Energy Economics and the Rational Use of Energy, University of Stuttgart, Stuttgart, Germany

**Joseph Gardella Jr.**, Ph.D., Professor and Larkin Chair of Chemistry, University at Buffalo, State University of New York, Buffalo, NY, USA

**Philip Germansderfer**, D.Sc., International Marketing Sales, Fluid Managment Systems, Watertown, MA, USA

**Gillian Gibson**, M.Sc., Environmental Scientist, Gibson Consulting and Training, Cheshire, United Kingdom

**Andreas Gies**, Ph.D., Director and Professor, Department for Environmental Hygiene, Federal Environment Agency, Berlin, Germany

**Robert Gould**, M.D., President, San Francisco Bay Area Chapter of Physicians for Social Responsibility, Berkeley, CA, USA

**Konstanze Grote**, Ph.D., Institute of Clinical Pharmacology and Toxicology, Charité University Medical School Berlin, Berlin, Germany

**Rui Guo**, Ministry of Environment, Toronto, Ontario, Canada

**Jana Hajslova**, Ph.D., Head of Department of Food Analysis, Institute of Chemical Technology, Prague, Czech Republic

**Ralph Hall**, Ph.D., Assistant Professor, Virginia Polytechnic Institute, Blacksburg, VA, USA

**Bruce Hammock**, Ph.D., Professor, Entomology, University of California, Davis, Davis, CA, USA

**Tran Thi Tuyet Hanh**, M.P.H., Lecturer in Environmental Health, Hanoi School of Public Health, Hanoi, Vietnam

**Kim Harley**, Ph.D., Associate Director, Center for Children’s Environmental Health Research, University of California, Berkeley, Berkeley, CA, USA

**Stuart Harrad**, Ph.D., Professor, Environmental Chemistry, University of Birmingham, Birmingham, United Kingdom

**Robert Harrison**, M.D., M.P.H., Clinical Professor, Occupational and Environmental Medicine, University of California, San Francisco, San Francisco, CA, USA

**Line Smastuen Haug**, Doctoral Student, Norwegian Institute of Public Health, Oslo, Norway

**Yasuhiro Hirai**, Ph.D., Associate Professor, Environment Preservation Engineering, Kyoto University, Kyoto, Japan

**Ivan Holoubek**, Ph.D., Director and Professor, Masaryk University, Research Centre for Toxic Compounds in the Environment, Brno, Czech Republic

**Ron Hoogenboom**, Ph.D., Toxicologist, RIKILT Institute of Food Safety, Wageningen University and Research Center, Wageningen, the Netherlands, and Board Member, International Panel on Chemical Pollution, Zürich, Switzerland

**David Hope**, CEO, Pacific Rim Laboratories, Surrey, British Columbia, Canada

**William J. Hirzy**, Ph.D., Chemist in Residence, American University, Washington, DC, USA

**Heinrich Huehnerfuss**, Ph.D., Professor, University of Hamburg, Hamburg, Germany

**Alastair Iles**, Ph.D., Assistant Professor, Environmental Science, Policy, and Management, University of California, Berkeley, Berkeley, CA, USA

**Tomohiko Isobe**, Ph.D., Senior Research Fellow, Ehime University, Matsuyama City, Japan

**Kristina Jakobsson**, Ph.D., Associate Professor, Occupational and Environmental Medicine, Lund University, Lund, Sweden

**Sarah Janssen**, M.D., Ph.D., M.P.H., Senior Scientist, Natural Resources Defense Council, New York City, NY, USA

**Niklas Johansson**, Scientist, Karolinska Institute, Stockholm, Sweden

**Catherine Karr**, M.D., Ph.D., M.S., Assistant Professor and Director, Pediatric Environmental Health Specialty Unit, Pediatrics, University of Washington, Seattle, WA, USA

**Donald Kennedy**, Ph.D., Bing Professor of Environmental Science, Emeritus, Stanford University, Palo Alto, CA, USA, and Editor Emeritus, *Science*

**Sergio Kuriyama**, Ph.D., Guest Scientist, Laboratory of Environmental Toxicology, National School of Public Health, Fiocruz, Brazil

**James Leckie**, M.S., Ph.D., C.L. Peck, Class of 1906 Professor of Engineering and Director, Center for Sustainable Development and Global Competitiveness, Stanford University, Palo Alto, CA, USA

**Pamela Lein**, Ph.D., Professor, Molecular Biosciences, University of California, Davis, Davis, CA, USA

**Juliana Leonel**, Ph.D., Postdoctoral Researcher, Universidade Federal do Rio Grande, Rio Grande, Brazil

**Mark Levine**, Ph.D., Leader, China Energy Group, and Former Director, Environmental Energy Technologies Division, Lawrence Berkeley National Laboratory, Berkeley, CA, USA

**Donald Lucas**, Ph.D., Deputy Director, Environment, Health, and Safety Division, Lawrence Berkeley National Laboratory, Berkeley, CA, USA

**Richard Luthy**, Ph.D., Silas H. Palmer Professor, Civil and Environmental Engineering, Stanford University, Palo Alto, CA, USA

**Karl Mair**, D.Sc., Senior Scientist, Eco Research SRL, Bolzano, Italy

**Govindan Malarvannan**, Ph.D., Research Fellow, Center for Marine Environmental Studies, Ehime University, Matsuyama City, Japan

**John Meeker**, M.S., Sc.D., Assistant Professor, Environmental Health Sciences, University of Michigan School of Public Health, Ann Arbor, MI, USA

**Richard Meigs**, P.E., Senior Principal Engineer, RJR Engineering, Ventura, CA, USA

**Mark Miller**, M.D., M.P.H., Director, Pediatric Environmental Health Specialty Unit, and Assistant Clinical Professor, Pediatrics, University of California, San Francisco, San Francisco, CA, USA

**Paolo Mocarelli**, M.D., Professor and Director, Department of Clinical Pathology, University of Milano Bicocca, Milano, Italy

**Rachel Morello-Frosch**, M.P.H., Ph.D., Associate Professor, Department of Environmental Science Policy and Management, University of California, Berkeley, Berkeley, CA, USA

**Jochen Mueller**, Ph.D., Professor, University of Queensland, Brisbane, Australia

**Tom Muir**, M.S., Retired, Environment Canada, Québec City, Quebec, Canada

**Martin Mulvihill**, Ph.D., Associate Director for Education and Outreach, Center for Green Chemistry, University of California, Berkeley, Berkeley, CA, USA

**Anbu Munasamy**, M.S., Ph.D., National Institute for Interdisciplinary Science and Technology–Council of Scientific and Industrial Research, Thiruvananthapuran, Kerala, India

**Richard Murphy**, Ph.D., Director of Science and Education, Jean-Michel Cousteau Ocean Futures Society, Santa Barbara, CA, USA

**Takeshi Nakano**, Ph.D., Research Professor, Center for Advanced Science and Innovation, Osaka University, Osaka, Japan

**Shoji Nakayama**, M.D., Ph.D., National Research Council Associate, U.S. Environmental Protection Agency, Washington, DC, USA

**Amgalan Natsagdorj**, Ph.D., Department Head, Environmental Chemistry, National University of Mongolia, Ulaanbaatar, Mongolia

**William Nazaroff**, Ph.D., Daniel Tellep Distinguished Professor and Vice Chair for Academic Affairs, Civil and Environmental Engineering, University of California, Berkeley, Berkeley, CA, USA

**John Neuberger**, Dr.Ph., M.P.H., M.B.A., Professor, Preventative Medicine and Public Health, University of Kansas School of Medicine, Kansas City, KS, USA

**Jessica Norrgran**, Doctoral Student, Stockholm University, Stockholm, Sweden

**Fardin Oliaei**, Ph.D., M.P.A., Consultant, Cambridge EnviroScience Consulting, LLC, Cambridge, MA, USA

**Kees Olie**, Ph.D., Associate Professor, University of Amsterdam, Amsterdam, the Netherlands

**Olaf Paepke**, Ph.D., Eurofins, Hamburg, Germany

**Victoria Persky**, M.D., Professor, University of Illinois at Chicago School of Public Health, Chicago, IL, USA

**Agneta Rannug**, Ph.D., Professor, Institute of Environmental Medicine, Karolinska Institute, Stockholm, Sweden

**Ulf Rannug**, Ph.D., Professor, Genetics, Microbiology and Toxicology, Stockholm University, Stockholm, Sweden

**Eric Reiner**, Ph.D., Senior Research Scientist, Ontario Ministry of Environment, Toronto, Ontario, Canada

**Martin Reinhard**, Ph.D., Professor, Civil and Environmental Engineering, Stanford University, Palo Alto, CA, USA

**Karen Rice**, M.D., Physician, Obstetrics and Gynecology, Walnut Creek Kaiser, Walnut Creek, CA, USA

**Robert H. Rice**, Ph.D., Professor of Environmental Toxicology, University of California, Davis, Davis, CA, USA

**Anthony Roach**, Ph.D., Senior Research Scientist, Government of New South Wales, Sydney, Australia

**David Roberts**, Ph.D., William R. Kenan, Jr. Professor of Astrophysics, Brandeis University, Waltham, MA, USA

**Mary Roberts**, Ph.D., Professor, Chemistry, Boston College, Boston, MA, USA

**Christina Ruden**, Ph.D., Professor, Philosophy and the History of Technology, Royal Institute of Technology, Stockholm, Sweden

**Cindy Lee Russell**, M.D., Vice President of Community Health, Santa Clara County Medical Association, San Jose, CA, USA

**Kenneth Sauer**, Ph.D., Professor Emeritus of Chemistry, University of California, Berkeley, Berkeley, CA, USA

**Arnold Schecter**, M.D., M.P.H., Professor, Environmental and Occupational Health Sciences, University of Texas School of Public Health, Dallas, TX, USA

**Martin Scheringer**, D.Sc., Senior Scientist, ETH Zürich, Zürich, Switzerland, and Board Member, International Panel on Chemical Pollution, Zürich, Switzerland

**Ted Schettler**, M.D., M.P.H., Science Director, Science and Environmental Health Network, Ames, IA, USA

**Karl-Werner Schramm**, Ph.D., Professor and Chair, German Research Center for Environmental Health, Neuherberg, Germany

**Megan Schwarzman**, M.D., M.P.H., Research Scientist, University of California, Berkeley, Berkeley, CA, USA, and Associate Physician, University of California, San Francisco, San Francisco, CA, USA

**Susan D. Shaw**, Dr.PH., Director, Marine Environmental Research Institute, Blue Hill, ME, USA

**Heather Stapleton**, Ph.D., Assistant Professor, Nicholas School of the Environment, Duke University, Durham, NC, USA

**Kristina Sundqvist**, Ph.D., Project Assistant, Chemistry, Umeå University, Umeå, Sweden

**Patrice Sutton**, M.P.H., Research Scientist, Program on Reproductive Health and the Environment, University of California, San Francisco, San Francisco, CA, USA

**Shanna Swan**, Ph.D., Professor and Associate Chair for Research, Obstetrics and Gynecology, and Director, Center for Reproductive Epidemiology, University of Rochester School of Medicine, Rochester, NY, USA

**Takumi Takasuga**, Ph.D., Director, Shimadzu Techno-Research Inc., Kyoto, Japan

**Chris Talsness**, D.V.M., Working Group Leader in Reproductive Toxicology, Charite Universitatsmedizin Berlin, Berlin, Germany

**Cathrine Thomsen**, Ph.D., Senior Scientist, Norwegian Institute of Public Health, Oslo, Norway

**Gregg Tomy**, Ph.D., Adjunct Assistant Professor, Fisheries and Oceans, University of Manitoba, Winnipeg, Manitoba, Canada

**Joao Paulo Machado Torres**, Sc.D., Associate Professor, Universidade Federal do Rio de Janeiro, Rio de Janeiro, Brazil

**James Trosko**, Ph.D., Professor, Pediatrics and Human Development, Center for Integrative Toxicology, Michigan State University, East Lansing, MI, USA

**Mary Turyk**, Ph.D., M.P.H., Research Assistant Professor, University of Illinois at Chicago, Chicago, IL, USA

**Gunther Umlauf**, Ph.D., European Commission Joint Research Center, Ispra, Italy

**Bryan Vining**, Ph.D., Analytical Perspectives, Wilmington, NC, USA

**Qiuquan Wang**, Ph.D., Professor of Chemistry, Xiamen University, Xiamen, China

**Yawei Wang**, Ph.D., Research Center for Eco Environmental Science, Beijing, China

**Julie Shu-Li Wang**, Ph.D., Investigator, National Health Research Institute, Taipei, Taiwan

**Rosemary Waring**, Ph.D., Honorary Reader, Human Toxicology, University of Birmingham, Birmingham, United Kingdom

**Thomas F. Webster**, D.Sc., Associate Professor and Associate Chair, Department of Environmental Health, Boston University School of Public Health, Boston, MA, USA

**Charles Weschler**, Ph.D., Adjunct Professor, UMDNJ–Robert Wood Johnson Medical School and Rutgers University, New Brunswick, NJ, USA, and Continuing Visiting Professor, International Centre for Indoor Environment and Energy, Technical University of Denmark, Lyngby, Denmark

**Stevie Wilding**, Chemist, U.S. Environmental Protection Agency, Region 3, Philadelphia, PA, USA

**Duane Wilding**, M.E., Senior Engineer, Maryland Environmental Service, Millersville, MD, USA

**Gayle Windham**, Ph.D., Researcher, Breast Cancer and the Environment Research Centers, Research Triangle Park, NC, USA

**Tracey Woodruff**, Ph.D., M.P.H., Associate Professor and Director, Program on Reproductive Health and the Environment, University of California, San Francisco, San Francisco, CA, USA

**Jae-Ho Yang**, M.D., M.P.H., Professor, Catholic University of Daegu, Gyeongsan, Korea

**Tom Young**, M.P.P., Ph.D., Professor, Civil & Environmental Engineering, University of California, Davis, Davis, CA, USA

**Bin Zhao**, Doctoral Student, Environmental Toxicology, University of California, Davis, Davis, CA, USA

**R. Thomas Zoeller**, M.A., Ph.D., Professor, Biology Department, University of Massachusetts, Amherst, Amherst, MA, USA

**Ami Zota**, Sc.D., Postdoctoral Scholar, Program on Reproductive Health and the Environment, University of California, San Francisco, Oakland, CA, USA

## Supplemental Material

(212 KB) PDF Abbreviations and an Annotated Statement

