# Supplementary material for: San Antonio Statement on Brominated and Chlorinated Flame Retardants
Source: Environ Health Perspect. 2010 Oct 28;118(12):A516–8. doi: 10.1289/ehp.1003089 (PMC3002202; doi:10.1289/ehp.1003089)
Supplement: (212 KB) PDF — Abbreviations and an Annotated Statement [file ehp.1003089.s001.pdf]

Supplemental Material

San Antonio Statement on Brominated and Chlorinated Flame Retardants

Joseph DiGangi,[1] Arlene Blum,[2,3] Åke Bergman,[4] Cynthia A. de Wit,[5] Donald Lucas,[6] David Mortimer,[7] Arnold Schecter,[8] Martin Scheringer,[9] Susan D. Shaw,[10] and Thomas F. Webster[11]

[1]International POPs Elimination Network, Berkeley, California, USA; [2]Department of Chemistry, University of California, Berkeley, California, USA; [3]Green Science Policy Institute, Berkeley, California, USA; [4]Department of Materials and Environmental Chemistry, and [5]Department of Applied Environmental Science, Stockholm University, Stockholm, Sweden; [6]Lawrence Berkeley National Laboratory, Berkeley, California, USA; [7]Food Standards Agency, London, United Kingdom; [8]University of Texas School of Public Health, Dallas, Texas, USA; [9]Institute for Chemical and Bioengineering, ETH Zürich, Zürich, Switzerland; [10]Marine Environmental Research Institute, Center for Marine Studies, Blue Hill, Maine, USA; [11]Department of Environmental Health, Boston University School of Public Health, Boston, Massachusetts, USA

Abbreviations (page 2) and an Annotated Statement (page 3) are presented herein.

Abbreviations

Brominated and chlorinated flame retardants may be mentioned under different names and abbreviations. Preferred abbreviations, alternative abbreviations, chemical name and Chemical Abstract System (CAS) numbers , related to the compound names, are given for those compounds discussed in the "Annotated San Antonio Statement on Brominated and Chlorinated Flame Retardants" are listed below.

TBP-AE or ATT: 2,4,6-tribromophenyl allyl ether; CAS 3278-89-5

BTBPE: 1,2-Bis(2,4,6-tribromophenoxy)ethane; CAS 37853-59-1

BEHTBP: bis(2-ethylhexyl) tetrabromophthalate; CAS 26040-51-7

BTBPIE: 1,2-Bis(tetrabromophthalimido)ethane; CAS 32588-76-4

DBDPE: Decabromodiphenylethane; CAS 84852-53-9

DBHC-TCTD or HCDBCO: 5,6-Dibromo-1,10,11,12,13,13-hexachloro-11-tricyclo[8.2.1.02,9]tridecene; CAS 51936-55-1

DP: Dechlorane Plus, Bis (hexachlorocyclopentadieno)cyclooctane; CAS 13560-89-9

TBP-DBPE: 2,4,6-Tribromophenyl 2,3-dibromopropyl ether; CAS 35109-60-5

HBB: Hexabromobenzene; CAS 87-82-1

HBCDD[1] or HBCD: Hexabromocyclododecane; CAS 3194-55-6; Major isomers are: α-, β-and γ-HBCDD

PBEB: Pentabromoethylbenzene; CAS 85-22-3

PBT: Pentabromotoluene; CAS 87-83-2

POPs: Persistent Organic Pollutants

SCCP: Short-chain chlorinated paraffins; CAS 85535-84-8 and 71011-12-6

EH-TBB or TBB: 2-Ethylhexyl-2,3,4,5-tetrabromobenzoate; CAS 183658-27-7

TBBPA: Tetrabromobisphenol A; CAS 79-94-7

TBBPA-DAE; Tetrabromobisphenol A diallyl ether; CAS 25327-89-3

TBBPA-DBPE: Tetrabromobisphenol A bis(2,3-dibromopropyl) ether; CAS 21850-44-2

TBECH: 1,2-Dibromo-4-(1,2-dibromoethyl)cyclohexane; CAS 3322-93-8

DEHTBP or TBPH: Di(2-ethylhexyl) tetrabromophthalate; CAS 26040-51-7

TCEP: Tris(2-chloroethyl) phosphate; CAS 115-96-8

TDCPP or TDCP: Tris(1,3-dichloroisopropyl) phosphate; CAS 13674-87-8

[1] HBCDD is used herein to distinguish hexabromocyclododecane from hexabromocyclodecane (CAS 25495-98-1) for which HBCD is also used as an abbreviation as well.

Annotated San Antonio Statement on Brominated and Chlorinated Flame Retardants

1. Parties to the Stockholm Convention have taken action on three brominated flame retardants that have been listed in the treaty for global elimination. These substances include components of commercial pentabromodiphenyl ether and commercial octabromodiphenyl ether, along with hexabromobiphenyl. Another brominated flame retardant, hexabromocyclododecane, is under evaluation.
Commercial pentabromodiphenyl[2] ether (PentaBDE) has been commonly used in foam for furniture and commercial octabromodiphenyl ether[3] (OctaBDE) has been used in plastics for electronic products. Both substances have been listed in the Stockholm Convention on Persistent Organic Pollutants for prohibition of production, use, import and export in more than 170 countries (UNEP 2009). POPs pose a threat to Arctic ecosystems and health of indigenous communities that are particularly at risk because of the biomagnification of persistent organic pollutants and the contamination of their traditional foods (AMAP Assessment 2009).

Hexabromobiphenyl (CAS 36355-01-8) is a component of commercial polybrominated biphenyls (PBB), another halogenated flame retardant previously used in plastics for electrical products and foam for auto upholstery. It is also a POP and has been listed in the Stockholm Convention on Persistent Organic Pollutants for prohibition of production, use, import and export in more than 170 countries (UNEP 2009).

The Stockholm Convention POPs Review Committee is currently evaluating commercial hexabromocyclododecane (CAS 25637-99-4 and 3194-55-6), a brominated flame retardant frequently used in building materials, for possible addition to the Convention due to concerns about its persistence, bioaccumulation, long-range transport, and toxicity (UNEP/POPS/POPRC 2009a).

2. Many commonly-used brominated and chlorinated flame retardants can undergo long-range environmental transport.
Modeling studies have identified 120 high production volume brominated and chlorinated chemicals which are structurally similar to known Arctic contaminants and/or have partitioning properties that suggest they are potential Arctic contaminants (Brown and Wania 2008). These substances include the following halogenated flame retardants: tetra- to octabromodiphenyl ether, decabromodiphenyl ether, hexabromocyclododecane, tetrabromocyclohexane, chlorendic acid, tetrabromophthalic anhydride, and 2,4,6-tribromophenol.

Monitoring studies show that many brominated and chlorinated flame retardants are found in the Arctic or Antarctic indicating long-range transport. These include the following brominated and

---

[2] Tetrabromodiphenyl ether (CAS 40088-47-9), pentabromodiphenyl ether (CAS 32534-81-9 and other tetra- and pentabromodiphenyl ethers present in commercial pentabromodiphenyl ether

[3] Hexabromodiphenyl ethers BDE-153 (CAS 68631-49-2), BDE-154 (CAS 207122-15-4), heptabromodiphenyl ethers BDE-175 (CAS 446255-22-7), BDE-183 (CAS 207122-16-5), and other hexa- and heptabromodiphenyl ethers present in commercial octabromodiphenyl ether.

chlorinated flame retardants: components of Firemaster 550 (EH-TBB and BEHTBP) (Sagerup et al. 2010), Dechlorane Plus (Sverko et al. 2010), BEHTBP (Sagerup et al. 2010), BTBPE (Verreault et al. 2007a), DBDPE (Sagerup et al. 2010), TBECH (Tomy et al. 2008), HBCDD and PBEB (de Wit et al. 2010), SCCPs (Tomy et al. 1998), TBBPA (de Wit et al. 2010; SAICM 2009; Xie et al. 2007), TCEP (Laniewski et al. 1998), BEHTBP (Sagerup et al. 2010), and HBB (de Wit et al. 2010). The references are given as examples and not as a comprehensive list.

3. Many brominated and chlorinated flame retardants appear to be persistent and bioaccumulative, resulting in food chain contamination, including human milk.
Modeling studies examined 22,263 commercial substances that are not currently part of contaminant measurement programs identified 610 substances that are likely to be persistent and bioaccumulative (Howard and Muir 2010). These substances include the following flame retardants: TBP-AE, BTBPE, BEHTBP, BTBPIE, DBDPE, Dechlorane Plus, HBCDD, PBEB, TBBPA, TBBPA-DAE, TBBPA-DBPE, TBECH and TDCPP.

Monitoring studies show that many brominated and chlorinated flame retardants are found in the bodies of wildlife and humans, and some are found in the indoor environment. These include the following flame retardants: Firemaster 550 compounds, EH-TBB and BEHTBP, in house dust (Stapleton et al. 2008), in dolphins and porpoises (Lam et al. 2009), chlorinated tris (TDCPP) in indoor house dust (Stapleton et al. 2008), Dechlorane Plus in Great Lakes fish, herring gull eggs, and house dust (Gauthier et al. 2007; Hoh et al. 2006; Zhu et al. 2007), BTBPE in northern fulmar eggs, herring gull eggs and glaucous gulls in the Norwegian Arctic, house dust (Gauthier et al. 2007; Karlsson et al. 2006; Stapleton et al. 2008; Verreault et al. 2007a), DBDPE in fish and house and air craft dust (Law et al. 2006; Stapleton et al. 2008; Bergman et al. 2010), TBECH in beluga whales in the Canadian Arctic (Tomy et al. 2008), HBCDD in Arctic biota including polar bears, human serum, indoor dust, fish, breast milk (Fängström et al. 2008; Kakimoto et al. 2008; Letcher et al. 2009; Stapleton et al. 2008; Thomsen et al. 2010), DBHC-TCTD in house dust (Zhu et al. 2008), PBEB in herring gull eggs and glaucous gulls in the Norwegian Arctic (Gauthier et al. 2007; Verreault et al. 2007a), SCCPs in Arctic biota and breast milk (Thomas et al. 2006; Environment Canada 2004), TBBPA in marine mammals, predatory bird eggs, breast milk, umbilical cord serum, blood and adipose tissue (Cariou et al. 2008; Antignac et al. 2008; Jakobsson et al. 2002; Johnson-Restrepo et al. 2008; NTP 2002), and HBB in falcon eggs, eggs of Great Lakes gulls, glaucous gulls in the Norwegian Arctic, human serum (Gauthier et al. 2009; Verreault et al. 2007b; Zhu et al. 2009).

4. Many brominated and chlorinated flame retardants lack adequate toxicity information and the available data raises concerns.
In the US in the 1970s, brominated tris (tris(2,3-dibromopropyl) phosphate) was banned (U.S.Consumer Product Safety Commission 1977) from children's pajamas and chlorinated tris was removed from pajamas because these two flame retardants caused genetic mutations in the Ames test and were suspected carcinogens (Blum and Ames 1977; Gold et al. 1978). According to the US Consumer Product Safety Commission, chlorinated tris is a probable human carcinogen (Babich 2006). Dechlorane Plus is poorly characterized toxicologically though it shares the chlorinated norbornene moiety with dieldrin, chlordane, heptachlor, endrin – all substances listed in the Stockholm Convention (UNEP 2001), and endosulfan (under evaluation for the Stockholm Convention) (UNEP/POPS/POPRC 2009a). A metabolite of BTBPE is 2,4,6-tribomophenol, a thyroid disrupting chemical (Hamers et al. 2006; Suzuki et al. 2008) which has been found in

umbilical cord blood (Hovander et al. 2002; Kawashiro et al. 2008). DBDPE is structurally very similar to decaBDE but has not been assessed toxicologically. Neonatal exposure to decaBDE causes changes in learning and behavior in adult animals and an altered response to nicotine, indicating a change in the brain cholinergic system (Viberg et al. 2003; Viberg et al. 2007). TBECH is a strong androgen agonist (Larsson et al. 2006) that is mutagenic to mammalian cells *in vitro* (McGregor et al. 1991). HBCDD is very toxic to aquatic organisms and can disrupt the hypothalamic-pituitary-thyroid (HPT) axis, disrupting normal development, affecting the central nervous system, and inducing reproductive and developmental effects in mammals with some of them being trans-generational (European Commission 2008; Swedish Chemicals Agency 2009). DBHC-TCTD is poorly characterized toxicologically though the substance shares the chlorinated norbornene moiety with dieldrin, chlordane, heptachlor, endrin – all substances listed in the Stockholm Convention (UNEP 2001) – and endosulfan (under evaluation for the Stockholm Convention) (UNEP/POPS/POPRC 2009a). PBEB is poorly characterized toxicologically but the substance is a brominated analogue of ethylbenzene, a carcinogen. SCCPs are considered cancer causing under California's Safe Drinking Water and Toxic Enforcement Act of 1986, also known as Proposition 65 (OEHHA 1986). TBBPA is structurally similar to thyroxine and shows thyroid hormone activity *in vivo* and *in vitro* (Van der Ven et al. 2008). It shows estrogenic activity in animals (Kitamura et al. 2005) and inhibits neurotransmitter uptake affecting dopamine, GABA, and glutamate (Mariussen and Fonnum 2003). TCEP causes adverse reproductive outcomes (Beth-Hübner 1999; EHRT 1999) and is considered a carcinogen under California Office of Environmental Health hazard Assessment Proposition 65 (OEHHA 1986).

5. Many different types of brominated and chlorinated flame retardants have been incorporated into products even though comprehensive toxicological information is lacking. These products include foam used in furniture, plastics used in electrical and electronic products, building materials, textiles, and other types of products. For example:

PentaBDE: polyurethane foam used in upholstered furniture, carpet padding, and automobiles; polyurethane foam containing pentaBDE also is being reused in re-bonded carpet cushion and could be used in other recycled products (Daley et al. 2010).

OctaBDE: primarily used in acrylonitrile-butadiene-styrene (ABS) polymers for office electrical equipment; other uses include high impact polystyrene (HIPS), polybutylene terephthalate (PBT) and polyamide polymers (UNEP/POPS/POPRC 2009a).

DecaBDE: primarily used in high impact polystyrene (HIPS) for televisions, printers, and other electrical equipment; also used in thermoplastic polyesters, nylon, polypropylene and polyethylene for wires, cables, connectors and switches (Danish EPA 2006).

DEHTBP and EH-TBB: components of Firemaster 550, are e.g. used as a plasticizer for PVC (Harju et al 2008) and in wire and cable insulation, film and sheeting, carpet backing, coated fabrics, wall coverings and adhesives (OEHHA 2008).

Dechlorane Plus: used in electrical wires, cables, computer connectors, and plastic roofing (OEHHA 2008).

BTBPE: substitute for octaBDE (OEHHA 2008).

DBDPE: substitute for decaBDE (OEHHA 2008).

TBECH: used in polystyrene home insulation, adhesives in fabric and vinyl, electrical cables, plastic parts of appliances, and construction materials (OEHHA 2008).

HBCDD: used in polystyrene home insulation, in HIPS plastic for VCR housings and video cassettes, textile coating for upholstery fabric, bed mattresses, transportation upholstery, drapes, and wall coverings (OEHHA 2008).

DBHC-TCTD: used in polystyrene (OEHHA 2008).
PBEB: used in the 1970s and 1980s in polyester resins for circuit boards, textiles, adhesives, wire and cable coatings, polyurethanes and other resins (OEHHA 2008).
SCCPs: used for metal-working and cutting, flame retardants, and plasticizers in paint and sealants (OEHHA 2008).
TBBPA: used in printed circuit boards and various plastics and resins (OEHHA 2008).
TDCCP: used in polyurethane foam as a pentaBDE substitute, and in plastics, resins, and as a fabric back-coating (OEHHA 2008).
TCEP: used in polyurethane foam, plastics, carpet backing, and fabric back-coating (OEHHA 2008).

6. Brominated and chlorinated flame retardants present in a variety of products are released to the indoor and outdoor environments.
Most brominated and chlorinated flame retardant chemicals, including PBDEs, are additive flame retardants in that they are simply mixed with the polymer resin as plastics and foams are being made and are not chemically bound to the material. Consequently, these chemicals leach continuously out of the final product (Bergman 1989; de Wit 2002; Rahman et al. 2001; Bergman 2005). Over time, these chemicals accumulate in indoor air (Harrad et al. 2010) and eventually enter the natural environment (Hale et al. 2006; Moeckel et al. 2010). Given the ubiquity of these products in the modern world, it should come as no surprise that flame retardant chemicals are being found in all environmental matrices examined including air, water, soil sediment, and sewage sludge (de Wit et al. 2010; Harrad et al. 2009; Shaw and Kannan 2009).

7. Near-end-of-life and end-of-life electrical and electronic products are a growing concern as a result of dumping in developing countries, which results in the illegal transboundary movement of their hazardous constituents. These include brominated and chlorinated flame retardants.
The consensus Decision II/4D of more than 110 countries at the Second International Conference on Chemicals Management in 2009 uses this language to describe concerns over hazardous substances such as brominated and chlorinated flame retardants within the life cycle of electrical and electronic products (SAICM 2009).

8. There is a lack of capacity to handle electronic waste in an environmentally sound manner in almost all developing countries and countries with economies in transition, leading to the release of hazardous substances that cause harm to human health and the environment. These substances include brominated and chlorinated flame retardants.
The consensus Decision II/4D of more than 110 countries at the Second International Conference on Chemicals Management in 2009 uses this language to describe concerns over hazardous substances such as brominated and chlorinated flame retardants within the life cycle of electrical and electronic products (SAICM 2009).

9. Brominated and chlorinated flame retardants may increase fire toxicity, but their overall benefit in improving fire safety has not been proven.
The fire safety benefit of brominated and chlorinated flame retardants is questionable because they can increase the release of carbon monoxide, toxic gases, and soot which are the cause of most fire deaths and injuries (Stec and Hull 2010). For example, in one experiment, compared to untreated foam, pentaBDE-treated foam released approximately twice the amount of smoke (833

m^2/kg vs. 413 m^2/kg), seven times the amount of carbon monoxide (0.13 kg/kg vs. 0.018 kg/kg), and nearly 70 times the amount of soot (0.88 kg/kg vs. 0.013 kg/kg) but only provided three additional seconds before ignition compared to untreated foam (19 seconds vs. 16 seconds) (Jayakody et al. 2000). Also, the California furniture standard, California Department of Consumer Affairs Technical Bulletin 117 (TB 117 2000) on the flammability of foam inside furniture neither protects the foam from ignition nor reduces the severity of a fire, two measures of efficacy (Babrauskas 1983; Schuhmann and Hartzell 1989; Talley 1995). In applications where chemical flame retardants are considered for use, an investigation should address whether flame retardancy is needed (i.e. breast feeding pillows do not need flame retardancy) and if so, whether appropriate fire safety benefits may be obtained from using chemicals or techniques that do not present such serious potential adverse environmental and human health consequences. In some cases, reducing the sources of ignition can prevent fires without adding potentially hazardous chemicals to consumer products[4].

10. When brominated and chlorinated flame retardants burn, highly toxic dioxins and furans are formed.

When brominated and chlorinated flame retardants burn, high yields of toxic brominated-, chlorinated-, and bromo-chlorinated dioxins and furans are formed (Söderström and Marklund 2002; Weber and Kuch 2003; Wichmann et al. 2002). In fact, the total amounts of brominated dioxins/furans generated from polybrominated diphenyl ethers are estimated in the tons scale and are comparable in magnitude to the total global formed amounts of chlorinated dioxins and furans (Zennegg et al. 2009). Brominated dioxins have toxicities similar to their chlorinated counterparts in human cell lines, mammalian species, and other assays (Behnisch et al. 2003; Birnbaum et al. 2003; Matsuda et al. 2010; Olsman et al. 2007). In addition, brominated dioxin/furan contamination has been reported in humans, including human milk as well as in food and dust (Ashizuka et al. 2008; Choi et al. 2003; Jogsten et al. 2010; Kotz et al. 2005; Ma et al. 2009; Matsuda et al. 2010b; Rose and Fernandes 2010; Suzuki et al. 2006; Suzuki et al. 2010; Takigami et al. 2008). State of the art incinerators have been used for disposal of flame retardant-containing materials. However an investigation of the process for disposing electronic waste containing halogenated flame retardants revealed that high levels of chlorinated, brominated-chlorinated and brominated dioxins and furans can be formed in the primary combustion zone (Hunsinger et al. 2002; UNEP/POPS/POPRC 2010). A secondary combustion zone can help destroy most of these unintentionally formed substances (Hunsinger et al. 2002). This and other studies indicate that combusting waste containing brominated and/or chlorinated flame retardants requires state-of-the-art incinerators operating under stringent conditions. Continuous or near-continuous monitoring of stack gases and frequent monitoring of residues is necessary to ensure that toxic contaminants are not released to the environment.

---

[4] In the US, California's flammability standard TB117 has led to the use of flame retardants in California furniture for more than thirty years. Despite this, an analysis of fire data from 1980 to 2005 by the National Fire Protection Association (NFPA) does not show a greater reduction in the rate of fire deaths in California compared to that of other states without such a standard. (Hall JR. US Unintentional Fire Death Rates by State. National Fire Protection Association (NFPA), Quincy, MA. 2008.) A 60% decrease in fire deaths in the United States since 1980 parallels the decrease in per capita cigarette consumption. Increased enforcement of improved building, fire, and electrical codes and the increased use of smoke detectors and sprinkler systems in new construction have also contributed to an increase in fire safety. In the US, an estimated 65% of reported home fire deaths in 2000-2004 resulted from fires in homes without working smoke alarms.

11. Brominated and chlorinated flame retardants as classes of substances are a concern for persistence, bioaccumulation, long-range transport, and toxicity.
Please see paragraphs 2-4 above.

12. There is a need to improve the availability of and access to information on brominated and chlorinated flame retardants and other chemicals in products in the supply chain and throughout each product's life cycle.
The consensus Decision II/4C of more than 110 countries at the Second International Conference on Chemicals Management in 2009 uses this statement to apply to all chemical substances (SAICM 2009).

13. Consumers can play a role in the adoption of alternatives to harmful flame retardants if they are made aware of the presence of the substances, for example, through product labeling.
This is the conclusion of the Stockholm Convention POPs Review Committee, an expert committee of the Convention that approved a guidance document on considerations relating to alternatives and substitutes (UNEP/POPS/POPRC 2009b).

14. The process of identifying alternatives to flame retardants should include not only alternative chemicals but also innovative changes in the design of products, industrial processes, and other practices that do not require the use of any flame retardant.
This is the conclusion of the Stockholm Convention POPs Review Committee, an expert committee of the Convention that approved a guidance document on considerations relating to alternatives and substitutes (UNEP/POPS/POPRC 2009b).

15. Efforts should be made to ensure that current and alternative chemical flame retardants do not have hazardous properties, such as mutagenicity and carcinogenicity, or adverse effects on the reproductive, developmental, endocrine, immune, or nervous systems.
This is the conclusion of the Stockholm Convention POPs Review Committee, an expert committee of the Convention that approved a guidance document on considerations relating to alternatives and substitutes (UNEP/POPS/POPRC 2009b).

16. When seeking exemptions for certain applications of flame retardants, the party requesting the exemption should supply information indicating why the exemption is technically or scientifically necessary and why potential alternatives are not technically or scientifically viable; a description of potential alternative processes, products, materials, or systems that eliminate the need for the chemical; and a list of sources researched.
These recommendations come from the Stockholm Convention POPs Review Committee, an expert committee of the Convention that approved a guidance document in 2009 on considerations relating to alternatives and substitutes for use by all Parties and Observers (UNEP/POPS/POPRC 2009a)

17. Wastes containing flame retardants with persistent organic pollutant (POP) characteristics, including products and articles, should be disposed of in such a way that the POP content is destroyed or irreversibly transformed so that they do not exhibit the characteristics of POPs.
Stockholm Convention Article 6, para1; in legal force for more than 170 countries (UNEP 2001).

18. Flame retardants with POP characteristics should not be permitted to be subjected to disposal operations that may lead to recovery, recycling, reclamation, direct reuse, or alternative uses of the substances.
Stockholm Convention Article 6, para1; in legal force for more than 170 countries (UNEP 2001).

19. Wastes containing flame retardants with POP properties should not be transported across international boundaries unless it is for disposal in such a way that the POP content is destroyed or irreversibly transformed.
Stockholm Convention Article 6, para1; in legal force for more than 170 countries (UNEP 2001).

20. It is important to consider product stewardship and extended producer responsibility aspects in the life-cycle management of products containing flame retardants with POP properties, including electronic and electrical products.
The consensus Decision II/4D of more than 110 countries at the Second International Conference on Chemicals Management in 2009 uses this statement to describe concerns over hazardous substances within the life cycle of electrical and electronic products (SAICM 2009).

References

AMAP Assessment. 2009. Human Health in the Arctic. Arctic Monitoring and Assessment Programme (AMAP), Oslo, Norway. 2009. 254 pp. Available: http://www.amap.no. file: Human_health-near_final.pdf [accessed 18 Oct. 2010].

Antignac JP, Cariou R, Maume D, Marchand P, Monteau F, Zalko D, Berrebi A, Cravedi JP, Andre F, Le Bizec B. 2008. Exposure assessment of fetus and newborn to brominated flame retardants in France: preliminary data. Mol Nutr Food Res 52:258-265.

Ashizuka Y, Nakagawa R, Hori T, Yasutake D, Tobiishi K, Sasaki K. 2008. Determination of brominated flame retardants and brominated dioxins in fish collected from three regions of Japan. Mol Nutr Food Res 52:273-283.

Babich MA. 2006. CPSC Staff Preliminary Risk Assessment of Flame Retardant (FR) Chemicals in Upholstered Furniture Foam. U S Consumer Product Safety Commission, Bethesda, MD. Available: www.cpsc.gov/library/foia/foia07/brief/ufurn2.pdf [accessed 15 Oct 2010].

Babrauskas V. 1983. Upholstered Furniture Heat Release Rates - Measurements and Estimation. Journal of Fire Sciences 1:9-32.

Behnisch PA, Hosoe K, Sakai S-I. 2003. Brominated dioxin-like compounds: in vitro assessment in comparison to classical dioxin-like compounds and other polyaromatic compounds. Environ Int 29:861-877.

Bergman Å. 1989. Brominated flame retardants in a global environmental perspective. Proceedings, Workshop on brominated aromatic flame retardants, Swedish National Chemicals Inspectorate, Solna, Sweden, 13-23.

Bergman Å. 2005. The Abysmal Failure of Preventing Human and Environmental exposure to Persitstent Brominated Flame Retardants: A Brief Historical Review of BRFs. In: Commemorating 25 years of Dioxin Symposia (Alaee M, Reiner E, Clement R, eds.). Toronto, Dioxin 2005, 32-40.

Bergman Å, Lindgren T, Smedje G, Jakobsson K, Athanassiadis I, Athanasiadou M, Meyer E. 2010. PBDEs and non-PBDEs in aircraft cabin and cockpit air and dust. Organohalogen Compd., Dioxin 2010, San Antonio.

Beth-Hübner M. 1999. Toxicological evaluation and classification of the genotoxic, carcinogenic, reprotoxic and sensitising potential of tris(2-chloroethyl)phosphate. Int Arch Occup Environ Health 72 (Suppl. 3):M17-M23.

Birnbaum LS, Staskal DF, Diliberto JJ. 2003. Health effects of polybrominated dibenzo-*p*-dioxins (PBDDs) and dibenzofurans (PBDFs). Environ Int 29:855-860.

Blum A, Ames BN. 1977. Flame-retardant additives as possible cancer hazards. Science 195:17-23.

Brown TN, Wania F. 2008. Screening chemicals for the potential to he persistent organic pollutants: A case study of Arctic contaminants. Environ Sci Technol 42:5202-5209.

Cariou R, Antignac JP, Zalko D, Berrebi A, Cravedi JP, Maume D, Marchand P, Monteau F, Riu A, Andre F, Le Bizec B. 2008. Exposure assessment of French women and their newborns to tetrabromobisphenol-A: Occurrence measurements in maternal adipose tissue, serum, breast milk and cord serum. Chemosphere 73:1036-1041.

Choi J, Fujimaki TS, Kitamura K, Hashimoto S, Ito H, Suzuki N, Sakai S, Morita M. 2003. Polybrominated dibenzo-p-dioxins, dibenzofurans, and diphenyl ethers in Japanese human adipose tissue. Environ Sci Technol 37:817-821.

Daley RE, Shaw SD, Birnbaum LS, Blum A. 2010. It´s all about penta: Informing decision-makers about the properties of penta-BDE and its replacements [Abstract]. 30th International Symposium on Halogenated Persistance Organic Pollutants (POPs). Available: www.xcdtech.com/dioxin2010/pdf/1635.pdf [accessed 15 Oct 2010].

Danish EPA. 2006. Deca-BDE and alternatives in electrical and electronic equipment, Environmental Project No, 1141 2006. Available: http://www2.mst.dk/Udgiv/publications/2007/978-87-7052-349-3/pdf/978-87-7052-350-9.pdf [accessed 12 Oct 2010].

de Wit C. 2002. An overview of brominated flame retardants in the environment. Chemosphere 46:583-624.

de Wit CA, Herzke D, Vorkamp K. 2010. Brominated flame retardants in the Arctic environment - trends and new candidates. Sci Total Environ 408:2885-2918.

EHRT. 1999. Final report on the reproductive toxicity of tris(2-chloroethyl)phosphate reproduction and fertility assessment in Swiss CD-1 mice when administered via gavage. Environmental Health Research and Testing Inc., Report Contract No. NTP.N01-ES-65142. in the order of National Toxicology program, NTIS/PB92-129170.

Environment Canada. 2004. Follow-up report on a PSL1 substance: Chlorinated Paraffins. Environment Canada. Available: http://www.ec.gc.ca/substances/ese/eng/psap/PSL1_chlorinated_paraffins.cfm [accessed 12 Oct 2010].

European Commission. 2008. Risk assessment hexabromocyclododecane, Final report May 2008. Available: http://ecb.jrc.ec.europa.eu/documents/Existing-Chemicals/RISK_ASSESSMENT/REPORT/hbcddreport044.pdf . [accessed 12 Oct 2010].

Fängström B, Athanassiadis I, Odsjö T, Noren K, Bergman Å. 2008. Temporal trends of polybrominated diphenyl ethers and hexabromocyclododecane in milk from Stockholm mothers, 1980-2004. Mol Nutr Food Res 52:187-193.

Gauthier LT, Hebert CE, Weseloh DVC, Letcher RJ. 2007. Current-use flaime retardants in the eggs of herring gulls (Larus argentatus) from the Laurentian Great lakes. Environ Sci Technol 41:4561-4567.

Gauthier LT, Potter D, Hebert CE, Letcher RJ. 2009. Temporal Trends and Spatial Distribution of Non-polybrominated Diphenyl Ether Flame Retardants in the Eggs of Colonial Populations of Great Lakes Herring Gulls. Environ Sci Technol 43:312-317.

Gold MD, Blum A, Ames BN. 1978. . Another flame retardant, tris-(1,3-Dichloro-2-Propyl)-phosphate, and its expected metabolites are mutagens. Science 200:785-787.

Hale RC, La Guardia MJ, Harvey E, Gaylor MO, Mainor TM. 2006. Brominated flame retardant concentrations and trends in abiotic media. Chemosphere 64:181-186.

Hamers T, Kamstra JH, Sonneveld E, Murk AJ, Kester MHA, Andersson PL, Legler J, Brouwer A. 2006. In Vitro Profiling of the Endocrine-Disrupting Potency of Brominated Flame Retardants. Toxicol Sci 92:157-173.

Harju M, Heimstad ES, Herzke D, Sandanger T, Posner S, Wania F. 2009. Current State of Knowledge and Monitoring requirements: Emerging "New" brominated flame retardants in flame retarded products and the environment. Norwegian Pollution Control Authority Report 2462. Available: http://www.klif.no/publikasjoner/2462/ta2462.pdf [accessed 17 Oct 2010].

Harrad S, de Wit C, Abdallah MA-E, Bergh C, Björklund JA, Covaci A, Darnerud PO, de Boer J, Diamond M, et al. 2010. Indoor contamination with hexabromocyclododecanes, polybrominated diphenyl ethers, and perfluoroalkyl compounds: An important exposure pathway for peoble? Environ Sci Technol 44:3221-3231.

Harrad S, Abdallah MAE, Rose NL, Turner SD, Davidson TA, Thomas A. 2009. Current-Use Brominated Flame Betardants in Water, Sediment, and Fish from English Lakes. Environ Sci Technol 43: 9077-9083. Addition/Correction in Environ Sci Technol 44: 5318.

Hoh E, Zhu LY, Hites RA. 2006. Dechlorane plus, a chlorinated flame retardant, in the Great Lakes. Environ Sci Technol 40:1184-1189.

Hovander L, Malmberg T, Athanasiadou M, Athanassiadis I, Rahm S, Bergman Å, Klasson Wehler E. 2002. Identification of hydroxylated PCB metabolites and other phenolic halogenated pollutants in human blood plasma. Arch Environ Contam Toxicol 42:105-117.

Howard PH, Muir DCG. 2010. Identifying New Persistent and Bioaccumulative Organics Among Chemicals in Commerce. Environ Sci Technol 44:2277-2285.

Hunsinger H, Jay K, Vehlow J. 2002. Formation and destruction of PCDD/F inside a grate furnace. Chemosphere 46:1263-1272.

Jakobsson K, Thuresson K, Rylander L, Sjödin A, Hagmar L, Bergman Å. 2002. Exposure to polybrominated diphenyl ethers and tetrabromobisphenol A among computer technicians. Chemosphere 46:709-716.

Jayakody C, Myers D, Sorathia U, Nelson GL. 2000. Fire-retardant characteristics of water-blown molded flexible polyurethane foam materials. J Fire Sci 18:430-455.

Jogsten IE, Hagberg J, Lindstrom G, van Bavel B. 2010. Analysis of POPs in human samples reveal a contribution of brominated dioxin of up to 15% of the total dioxin TEQ. Chemosphere 78:113-120.

Johnson-Restrepo B, Adams DH, Kannan K. 2008. Tetrabromobisphenol A (TBBPA) and hexabromocyclododecanes (HBCDs) in tissues of humans, dolphins, and sharks from the United States. Chemosphere 70:1935-1944.

Kakimoto K, Akutsu K, Konishi Y, Tanaka Y. 2008. Time trend of hexabromocyclododecane in the breast milk of Japanese women. Chemosphere 71:1110-1114.

Karlsson M, Ericson I, van Bavel B, Jensen JK, Dam M. 2006. Levels of brominated flame retardants in Northern Fulmar (Fulmarus glacialis) eggs from the Faroe Islands. Sci Total Environ 367:840-846.

Kawashiro Y, Fukata H, Omori-Inoue M, Kubonoya K, Jotaki T, Takigami H, Sakai Si, Mori C. 2008. Perinatal exposure to brominated flame retardants and polychlorinated biphenyls in Japan. Endocr J (Kyoto, Jpn) 55:1071-1084.

Kitamura S, Suzuki T, Sanoh S, Kohta R, Jinno N, Sugihara K, Yoshihara S, Fujimoto N, Watanabe H, Ohta S. 2005. Comparative Study of the Endocrine-Disrupting Activity of Bisphenol A and 19 Related Compounds. Toxicol Sci 84:249-259.

Kotz, A, Malisch, R, Kypke, K, Oehme, M. 2005. PBDE, PBDD/F and mixed chlorinated-brominated PXDD/F in pooled human milk samples from different countries. Organohalogens Compounds In Toronto, Canada 67:1540-1544.

Lam JCW, Lau RKF, Murphy MB, Lam PKS. 2009. Temporal Trends of Hexabromocyclododecanes (HBCDs) and Polybrominated Diphenyl Ethers (PBDEs) and Detection of Two Novel Flame Retardants in Marine Mammals from Hong Kong, South China. Environ Sci Technol 43:6944-6949.

Laniewski K, BorEn H, Grimvall A. 1998. Identification of Volatile and Extractable Chloroorganics in Rain and Snow. Environ Sci Technol 32:3935-3940.

Larsson A, Eriksson LA, Andersson PL, Ivarson P, Olsson PE. 2006. Identification of the Brominated Flame Retardant 1,2-Dibromo-4-(1,2-dibromoethyl)cyclohexane as an Androgen Agonist. J Med Chem 49:7366-7372.

Law K, Halldorson T, Danell R, Stern G, Gewurtz S, Alaee M, Marvin C, Whittle M, Tomy G. 2006. Bioaccumulation and trophic transfer of some brominated flame retardants in a Lake Winnipeg (Canada) food web. Environ Toxicol Chem 25:2177-2186.

Letcher RJ, Gebbink WA, Sonne C, Born EW, McKinney MA, Dietz R. 2009. Bioaccumulation and biotransformation of brominated and chlorinated contaminants and their metabolites in ringed seals (Pusa hispida) and polar bears (Ursus maritimus) from East Greenland. Environ Int 35:1118-1124.

Ma J, Addink R, Yun S, Cheng J, Wang W, Kannan K. 2009. Polybrominated Dibenzo-p-dioxins/Dibenzofurans and Polybrominated Diphenyl Ethers in Soil, Vegetation, Workshop-Floor Dust, and Electronic Shredder Residue from an Electronic Waste Recycling Facility and in Soils from a Chemical Industrial Complex in Eastern China. Environ Sci Technol 43:7350-7356.

Mariussen E, Fonnum F. 2003. The effect of brominated flame retardants on neurotransmitter uptake into rat brain synaptosmes and vesicles. Neurochemistry International 43:533-542.

Matsuda M, Okimoto M, Yousuke T, Nakamura M, Handa H, Kawano M, Nose Kea. 2010. Estimation of PBDD/DF toxicity equivalency factors from Ah receptor binding affinity and clearance rate in rat. Proceedings from the Fifth International Symposium on BFR, 2010, Kyoto, Japan Available: http://www.bfr2010.com/abstract-download/2010/90086.pdf. [accessed 15 Oct 2010].

McGregor DB, Brown AG, Howgate S, McBride D, Riach C, Caspary WJ. 1991. Responses of the L5178Y mouse lymphoma cell forward mutation assay. V: 27 coded chemicals. Environ Mol Mutagen 17:196-219.

Moeckel C, Gasic B, MacLeod M, Scheringer M, Jones KC, Hungerbühler K. 2010. Estimation of the source strength of polybrominated diphenyl ethers based on their diel variability in air in Zurich, Switzerland. Environ. Sci. Technol. 44:4225-4231.

NTP (National Toxicology Program). Tetrabromobisphenol A [79-94-7]. 2002. Review of Toxicological Literature. National Institute of Environmental Health Sciences . Available: http://ntp.niehs.nih.gov/ntp/htdocs/Chem_Background/ExSumPdf/tetrabromobisphenola.pdf [accessed 12 Oct 2010].

OEHHA (Office of Environmental Health Hazard Assessment). 1986. The Safe Drinking Water and Toxic Enforcement Act of 1986. Proposition 65 Available: http://www.oehha.org/prop65.html [accessed 16 Oct 2010].

OEHHA (Office of Environmental Health Hazard Assessment). 2008. Brominated and chlorinated organic chemical compounds used as flame retardants. OEHHA . Available: http://oehha.ca.gov/multimedia/biomon/pdf/FlameRetardants_FourMore.pdf [accessed 12 Oct 2010].

Olsman H, Engwall M, Kammann U, Klempt M, Otte J, Bavel Bv, Hollert H. 2007. Relative differences in aryl hydrocarbon receptor-mediated response for 18 polybrominated and mixed halogenated dibenzo-p-dioxins and -furans in cell lines from four different species. Environ Toxicol Chem 26:2448-2454.

Rahman F, Langford KH, Scrimshaw MD, Lester JN. 2001. Polybrominated diphenyl ether (PBDE) flame retardants. Sci Total Environ 275:1-17.

Rose M, Fernandes A. 2010. Are BFRs responsible for brominated dioxins and furans (PBDD/Fs) in blood? Proceedings from the Fifth International Symposium on BFR, 2010, Kyoto, Japan. Available: http://www.bfr2010.com/abstract-download/2010/90029.pdf. [accessed 15 Oct 2010].

Sagerup K, Herzke D, Harju M, Evenset A, Christensen GN, Routti H, Fuglei E, Aars J, Strom H, Gabrielsen GW. 2010. New brominated flame retardants in Arctic biota. Sci Total Environ 408:2885-2918.

SAICM (Strategic Approach to International Chemicals Management). 2009. Decision 11/4D of the Second International Conference on Chemicals Management. Available: http://www.saicm.org/documents/iccm/ICCM2/ICCM2%20Report/ICCM2%2015%20FINAL%20REPORT%20E.pdf [accessed 12 Oct 2010].

Schuhmann JG, Hartzell GE. 1989. Flaming combustion characteristics of upholstered furniture. J Fire Sci 7:386-402.

Shaw SD, Kannan K. 2009. Polybrominated diphenyl ethers in marine ecosystems of the American continents: foresight from current knowledge. Rev Environ Health 24:157-229.

Söderström G, Marklund S. 2002. PBCDD and PBCDF from incineration of waste - containing brominated flame retardants. Environ Sci Technol 36:1959-1964.

Stapleton HM, Allen JG, Kelly SM, Konstantinov A, Klosterhaus S, Watkins D, McClean MD, Webster TF. 2008. Alternate and new brominated flame retardants detected in US house dust. Environ Sci Technol 42:6910-6916.

Stec A, Hull R. 2010. Fire toxicity.Woodhead publishing Limited, Oxford.

Suzuki G, Nose K, Takigami H, Takahashi S, Sakai SI. 2006. PBDEs and PBDD/Fs in house and office dust from Japan. Organohalogen Compd 68:1843-1846.

Suzuki G, Someya M, Takahashi S, Takigami H, Sakai S, Tanabe S. 2010. Dioxin-like compounds in Japanese indoor dusts: Brominated dibenzofurans strongly contribute to dioxin-like activity evaluated by in vitro bioassay. Proceedings from the Fifth International Symposium on BFR, Kyoto, Japan. Available: http://www.bfr2010.com/abstract-download/2010/90062.pdf [accessed 15 Oct 2010].

Suzuki G, Takigami H, Watanabe M, Takahashi S, Nose K, Asari M, Sakai SI. 2008. Identification of brominated and chlorinated phenols as potential thyroid-disrupting compounds in indoor dusts. Environmental science & technology 42:1794-1800.

Sverko E, Harner T, Lee SC, McCarry BE. 2010. Dechlorane Plus in the Global Atmospheric Passive Sampling (GAPS) study. Organohalogen Compd., Dioxin 2010, San Antonio.

Swedish Chemicals Agency. 2009. Proposal for harmonised classification and labelling, Substance hexachlorocyclododecane. ECHA. Available: http://echa.europa.eu/doc/consultations/cl/clh_axvrep_sweden_CD001435-70.pdf. [accessed 12 Oct 2010].

TB 117. 2000. Requirements, test procedure and apparatus for testing the flame retardance of resilient filling materials used in upholstred furniture. State of California, Department of Consumer Affairs, Bureau of Home Furnishings and Thermal Insulation. Available: http://www.bhfti.ca.gov/industry/117.pdf [accessed 17 Oct. 2010].

Takigami H, Suzuki G, Hirai Y, Sakai Si. 2008. Transfer of brominated flame retardants from components into dust inside television cabinets. Chemosphere 73:161-169.

Talley TH. 1995. Phases 1&2, UFAC Small Open Flame Tests and Cigarette Ignition Tests. Proceedings from the Annual AFMA Flammability Conference. March 23, 1995.

Thomas GO, Farrar D, Braekevelt E, Stern G, Kalantzi OI, Martin FL, Jones KC. 2006. Short and medium chain length chlorinated paraffins in UK human milk fat. Environ Int 32:34-40.

Thomsen C, Stigum H, Frøshaug M, Broadwell SL, Becher G, Eggesbø M. 2010. Determinations of brominated flame retardants in breast milk from a large scale Norwegian study. Environ Int 36:68-74.

Tomy GT, Fisk AT, Westmore JB, Muir DCG. 1998. Environmental chemistry and toxicology of polychlorinated n-alkanes. Rev Environ Contam Toxicol 158:53-128.

Tomy GT, Pleskach K, Arsenault G, Potter D, McCrindle R, Marvin CH, Sverko E, Tittlemier S. 2008. Identification of the novel cycloaliphatic brominated flame retardant 1,2-dihromo-4-(1,2-dibromoethyl)cyclo-hexane in Canadian arctic beluga (Delphinapterus leucas). Environ Sci Technol 42:543-549.

U.S. Consumer Product Safety Commission. 1977. NEWS from CPSC - CPSC Bans TRIS-Treated Children's Garments. U.S.Consumer Product Safety Commission. Available: http://www.cpsc.gov/cpscpub/prerel/prhtml77/77030.html [accessed 12 Oct 2010].

UNEP (United Nations Environment Programme). 2001. Final act of the conference of plenipotentaries on the Stockholm convention on persistent organic pollutants. Available: http://www.chem.unep.ch/pops/ Stockholm, Sweden, United Nations Environment Programme. [accessed 12 Oct 2010].

UNEP (United Nations Environment Programme). 2009. Stockholm Convention text and annexes as amended in 2009. Available: http://chm.pops.int/Convention/tabid/54/language/en-US/Default.aspx#convtext [accessed 17 Oct 2010].

UNEP/POPS/POPRC. (Stockholm Convention Persistent Organic Pollutants Review Committee). 2009a. Proposal to list hexabromocyclododecane in Annex A of the Stockholm Convention on Persistent Organic Pollutants UNEP/POPS/POPRC.5/4 Available: http://chm.pops.int/Convention/POPs%20Review%20Committee/Chemicals/tabid/781/language/en-US/Default.aspx [accessed 17 Oct. 2010].

UNEP/POPS/POPRC. (Stockholm Convention Persistent Organic Pollutants Review Committee). 2009b. General guidance on considerations related to alternatives and substitutes for listed persistent organic pollutants and candidate chemicals. UNEP/POPS/POPRC.5/10/Add.1 Available: http://chm.pops.int/Convention/POPsReviewCommittee/POPRCMeetings/POPRC5/POPRC5ReportandDecisions/tabid/719/language/en-US/Default.aspx [accessed 17 Oct. 2010].

UNEP/POPS/POPRC. (Stockholm Convention Persistent Organic Pollutants Review Committee). 2010. Technical review of the implications of recycling commercial pentabromodiphenyl ether and commercial octabromodiphenyl ether. Stockholm Convention POPs Review Committee, UNEP/POPS/POPRC.6/INF/7. Available: http://chm.pops.int/Convention/POPsReviewCommittee/POPRCMeetings/POPRC6/POPRC6Documents/tabid/783/language/en-US/Default.aspx [accessed 17 Oct 2010].

Van der Ven LTM, van de Kuil T, Verhoef A, Verwer CM, Lilienthal H, Leonards PEG, Schauer UMD, Canton RF, Litens S, De Jong FH, Visser TJ, Dekant W, Stern N, Hakansson H, Slob W, van den Berg M, Vos JG, Piersma AH. 2008. Endocrine effects of tetrabromobisphenol-A (TBBPA) in Wistar rats as tested in a one-generation reproduction study and a subacute toxicity study. Toxicol 245:76-89.

Verreault J, Gebbink WA, Gauthier LT, Gabrielsen GW, Letcher RJ. 2007a. Brominated flame retardants in glaucous gulls from the Norwegian Arctic: More than just an issue of polybrominated diphenyl ethers. Environ Sci Technol 41:4925-4931.

Verreault J, Gebbink WA, Gauthier LT, Gabrielsen GW, Letcher RJ. 2007b. Brominated Flame Retardants in Glaucous Gulls from the Norwegian Arctic: More Than Just an Issue of Polybrominated Diphenyl Ethers. Environ Sci Technol 41:4925-4931.

Viberg H, Fredriksson A, Jakobsson E, Örn U, Eriksson P. 2003. Neurobehavioral derangements in adult mice recieving decabrominated diphenyl ether (PBDE 209) during a defined period of neonatal brain develpment. Toxicol Sci 76:112-120.

Viberg H, Fredriksson A, Eriksson P. 2007. Changes in spontaneous behavior and altered response to nicotine in the adult rat, after neonatal exposure to the brominated flame retardant, decabrominated diphenyl ether (PBDE 209). NeuroToxicology 28:136-142.

Weber R, Kuch B. 2003. Relevance of BFRs and thermal conditions on the formation pathways of brominated and brominated-chlorinated dibenzodioxins and dibenzofurans. Environ Int 29:699-710.

Wichmann H, Dettmer FT, Bahadir M. 2002. Thermal formation of PBDD/F from tetrabromobisphenol A- a comparison of polymer linked TBBP A with its additive incorporation in thermoplastics. Chemosphere 47:349-355.

Xie Z, Ebinghaus R, Lohmann R, Heemken O, Caba A, Puettmann W. 2007. Trace determination of the flame retardant tetrabromobisphenol A in the atmosphere by gas chromatography-mass spectrometry. Anal Chim Acta 584:333-342.

Zennegg, M, Yu, X, Wong, M, Weber, R. 2009. Fingerprints of chlorinated, brominated and mixed halogenated dioxins at two e-waste recycling sites in Guiyu/China. Organohalogen Compd 71, Dioxin 2010, Beijing.

Zhu J, Feng Yl, Shoeib M. 2007. Detection of Dechlorane Plus in Residential Indoor Dust in the City of Ottawa, Canada. Environ Sci Technol 41:7694-7698.

Zhu J, Hou Y, Feng Yl, Shoeib M, Harner T. 2008. Identification and Determination of Hexachlorocyclopentadienyl-Dibromocyclooctane (HCDBCO) in Residential Indoor Air and Dust: A Previously Unreported Halogenated Flame Retardant in the Environment. Environ Sci Technol 42:386-391.

Zhu L, Ma B, Hites RA. 2009. Brominated Flame Retardants in Serum from the General Population in Northern China. Environ Sci Technol 43:6963-6968.
